# Une colite à CMV révélant un lupus érythémateux systémique

**DOI:** 10.11604/pamj.2014.19.380.3411

**Published:** 2014-12-15

**Authors:** Imed Ben Ghorbel, Najeh Boussetta, Jalel Boubaker, Alia Zehani, Mounir Lamloum, Mohamed Habib Houman

**Affiliations:** 1 Service de Médecine Interne, Hôpital La Rabta, 1007 Tunis, Tunisie; 2 Service de Gastrologie Hôpital La Rabta, 1007 Tunis, Tunisie; 3 Service d‘anatomopathologie, Hôpital La Rabta, 1007 Tunis, Tunisie

**Keywords:** Cytomégalovirus, CMV, colite, lupus érythémateux, Cytomegalovirus, CMV, colitis, systemic lupus erythematosus

## Abstract

Le cytomégalovirus (CMV) est responsable d'infections souvent asymptomatiques chez les immunocompétents mais également d'infections graves chez les immunodéprimés notamment chez les patients lupiques. La réactivation du CMV au cours du lupus est une complication fréquente mais rarement inaugurale. Nous rapportons l'observation d'un patient ayant présenté une colite à CMV révélatrice d'un lupus érythémateux systémique. Le diagnostic a été retenu sur les données sérologiques, de la biopsie colique et la bonne évolution après un traitement par ganciclovir.

## Introduction

Le lupus érythémateux systémique (LES) représente un facteur de risque de survenue des infections virales en particulier à virus herpétiques [1]. L'infection à cytomégalovirus (CMV) au cours du LES est plus rare que les autres infections herpétiques et consiste habituellement à une réactivation du CMV due à l'immunodépression induite par la maladie ou le traitement corticoïde [1]. Par contre, l'infection inaugurale est peu décrite dans la littérature [2]. Nous rapportons une observation d'un patient atteint d'un lupus érythémateux systémique révélé par une colite à CMV.

## Patient et observation

**Figure 1:**
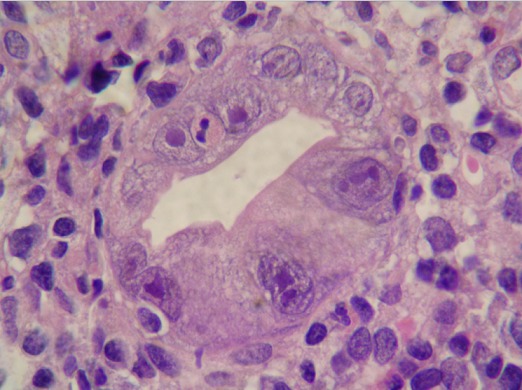
Étude anatomopathologique des biopsies coliques (HE x 400) révélant des inclusions intranucléaires en œil de hibou au niveau d'une glande de Luberkun

Il s'agit d'un patient âgé de 46 ans sans antécédent pathologique, ayant présenté une diarrhée glairosanglante évoluant depuis trois semaines associée à des rectorragies de moyenne abondance. Il se plaignait d'arthralgies inflammatoires fébriles. L'examen a révélé un patient maigre avec un poids de 52 Kg, une taille de 169 cm et un BMI de 18 kg/m2. La numération de la formule sanguine a montré une lymphopénie à 900 éléments/mm3 et une anémie normochrome normocytaire à 9 g/dl avec un test de coombs direct positif de type Ig G. Il existait un syndrome inflammatoire biologique avec une VS à 120 mm à la première heure et une CRP à 70 mg /l. A l’électrophorèse des protides, on notait un taux d'albumine à 17 g/l, une hyper alpha2 à 10 g/l et une hypergammaglobulinémie d'allure polyclonale à 20 g/l. Les gammaglutamyl transférase et les phosphatases alcalines étaient à 4 fois la normale. La radiographie du thorax a mis en évidence un épanchement pleural bilatéral et l’échographie cardiaque un épanchement péricardique de faible abondance. Les anticorps antinucléaires étaient positifs de type homogène à 13200 ainsi que les anti-DNA natifs. Les anticorps anti antigènes solubles, anti mitochondries et anti muscles lisses étaient absents. Les sérologies de l'hépatite B et C étaient négatives. La sérologie CMV était positive de type Ig M et Ig G avec un taux d'Ig G supérieur à 400 UI/ml. La coloscopie a objectivé une muqueuse colique congestive, érythémateuse et granitée, surmontée par plusieurs ulcérations superficielles sans intervalle de muqueuse saine. L’étude anatomopathologique des biopsies coliques a mis en évidence des lésions de cryptite et un revêtement renfermant des cellules à pseudo-inclusion éosinophile évocatrices d'une infection à CMV (Figure 1). Le diagnostic d'un lupus érythémateux systémique révélé par une colite à CMV a été retenu. Un traitement à base de ganciclovir à la dose de 10 mg/kg/j associé à de la prednisone à la dose de 1 mg/kg/j et de l'hydroxychloroquine à la dose de 200 mg/j a été instauré. L’évolution était marquée par la disparition de la diarrhée, de la pleurésie, des anomalies biologiques ainsi qu'une reprise de poids à raison de 3 kg en un mois. La coloscopie de contrôle était normale. Le recul actuel est de 40 mois avec aucune récidive de la diarrhée et une prise de poids estimée à 20 kg.

## Discussion

L'atteinte digestive, en particulier l'entérite, est une manifestation rare au cours du lupus érythémateux systémique dont la prévalence est estimée entre 0,2 et 1,1% des cas [3]. L'atteinte est classiquement jéjunale et/ou iléale observée dans 80 à 85% des cas alors que la colite est plus rare [3].

La présentation clinique de l'entérite lupique est variable. Elle peut mimer un tableau pseudochirurgical ou se manifester par une diarrhée glairosanglante [4]. Le diagnostic est surtout clinique et confirmé par la tomodensitométrie abdominale qui peut montrer un épaississement de la paroi de l'intestin grêle et du colon avec une prise de contraste, souvent associée à un épanchement péritonéal modéré et s'y ajoute le plus souvent une infiltration de la graisse mésentérique [5]. La coloscopie, lorsqu'elle est pratiquée, montre habituellement une paroi digestive œdématiée et des ulcérations [4]. Cette entérite est secondaire à une vascularite des artérioles et des veinules de la sous-muqueuse avec un dépôt de complexe immuns et de C3 associé à un infiltrat inflammatoire [6].

La réactivation des herpès virus est une complication fréquente au cours du lupus [7]. L'infection à CMV est plus rare que les autres infections herpétiques et revêt des localisations polymorphes [2]. Selon les données de la littérature, cinq des onze observations rapportaient des atteintes digestives. Il s'agissait d'une colite ulcérée dans deux cas [8, 9], d'une colite hémorragique [10], d'une œsophagite [11] et d'une péritonite par perforation iléale [12].

Le mécanisme de survenue de la colite à CMV est principalement lié à l'immunodépression induite par la maladie lupique mais également aux traitements corticoïdes et immunosuppresseurs en particulier par le cyclophosphamide administré pour les atteintes graves rénales et neurologiques [9]. Néanmoins, elle peut être inaugurale déclenchant alors la maladie [13] comme chez notre patient.

L'infection à CMV peut cependant mimer une poussée lupique rendant ainsi difficile, comme dans notre observation, de faire la part entre une colite survenant dans le cadre du lupus ou une colite à CMV. En effet, le diagnostic d'une primo-infection à CMV avec localisation colique parait assez plausible devant le tableau clinique initial associant une fièvre prolongée, la présence de pseudo-inclusions éosinophiles au niveau des cellules du revêtement épithélial, l'absence de lésion de vascularite, la sérologie CMV Ig M et Ig G positive ainsi que l’évolution favorable sous traitement antiviral et la disparition des lésions à la coloscopie de contrôle.

De même, l'association de 4 critères de l'ARA [14] (la pleuropéricardite, l'anémie auto-immune, lymphopénie, les AAN et les anti-DNA natifs) permettait également de retenir le diagnostic de lupus, bien que la positivité des AAN pouvait être aussi secondaire aux réactions immunologiques non spécifiques liées à l'infection aiguë à CMV, soit par l'hypergammaglobulinémie ou par l'hyperstimulation des lymphocytes B [14]. Néanmoins, la présence des anti-DNA plaide en faveur du lupus.

Contrairement à notre observation, particulière par le caractère inaugural de la colite à CMV concomitant au déclenchement d'une poussée de lupus, seules deux observations associant une colite ulcérée à CMV et un LES ont été rapportée à notre connaissance dans la littérature. Il s'agissait d'une patiente atteinte d'un lupus évoluant depuis cinq ans et traitée par des cures de cyclophosphamide pour une atteinte rénale, ayant présenté une infection virale à CMV et une colite ulcérée, mais contrairement à notre cas, sans mise en évidence d'inclusions virales à la biopsie [9]. Dans l'autre observation, le diagnostic de certitude de la colite ulcérée à CMV a été confirmé par l'immunomarquage de la pièce d'iléo-hémicolectomie en post-mortem [8].

## Conclusion

L'analyse des différentes observations de la littérature rapportant des infections aiguës à CMV révélatrices de LES ne permettent pas de conclure quant au rôle réel de ce virus dans l’étiopathogénie de la maladie lupique. Néanmoins, ces constatations devraient faire rechercher les marqueurs de l'infection à CMV à chaque découverte d'un LES et ce avant tout traitement immunosuppresseur car elle pourrait être responsable de l'exacerbation la maladie. De même, il parait indispensable de penser à la colite à CMV devant une diarrhée survenant chez un malade lupique.
